# Translocating bacteria in SIV infection are not stochastic and preferentially express cytosine methyltransferases

**DOI:** 10.1016/j.mucimm.2024.07.008

**Published:** 2024-07-31

**Authors:** Jacob K. Flynn, Alexandra M. Ortiz, Ivan Vujkovic-Cvijin, Hugh C. Welles, Jennifer Simpson, Fabiola M. Castello Casta, Debra S. Yee, Andrew R. Rahmberg, Kelsie L. Brooks, Marlon De Leon, Samantha Knodel, Kenzie Birse, Laura Noel-Romas, Anshu Deewan, Yasmine Belkaid, Adam Burgener, Jason M. Brenchley

**Affiliations:** a Barrier Immunity Section, Laboratory of Viral Diseases, NIAID, NIH, Bethesda, MD, USA; b Metaorganism Immunity Section, Laboratory of Host Immunity and Microbiome, NIAID, NIH, Bethesda, MD, USA; c Center for Global Health and Diseases, Case Western Reserve University, Cleveland, OH, USA; d Department of Obstetrics & Gynecology, University of Manitoba, Winnipeg, MB, Canada; e Integrated Data Sciences Section, Research Technologies Branch, NIAID, NIH, Bethesda, MD, USA; f Metaorganism Unit, Immunology Department, Institut Pasteur, 75724 Paris, France; g Department of Medicine Solna, Karolinksa Institutet, Stockholm, Sweden

## Abstract

Microbial translocation is a significant contributor to chronic inflammation in people living with HIV (PLWH) and is associated with increased mortality and morbidity in individuals treated for long periods with antiretrovirals. The use of therapeutics to treat microbial translocation has yielded mixed effects, in part, because the species and mechanisms contributing to translocation in HIV remain incompletely characterized. To characterize translocating bacteria, we cultured translocators from chronically SIV-infected rhesus macaques. Proteomic profiling of these bacteria identified cytosine-specific methyltransferases as a common feature and therefore, a potential driver of translocation. Treatment of translocating bacteria with the cytosine methyltransferase inhibitor decitabine significantly impaired growth for several species in vitro. In rhesus macaques, oral treatment with decitabine led to some transient decreases in translocator taxa in the gut microbiome. These data provide mechanistic insight into bacterial translocation in lentiviral infection and explore a novel therapeutic intervention that may improve the prognosis of PLWH.

## Background

Progressive HIV infection acquired in people living with HIV (PLWH) over time leads to the development of chronic inflammation that persists despite fully suppressed viremia on antiretroviral (ARV) therapy.^1^ In ARV-treated people living with HIV (PLWH) − particularly those that started ARVs years after HIV acquisition − residual inflammation is associated with non-HIV comorbidities including cardiovascular disease, neurological disorders, non AIDS-defining cancers, and increased mortality.^2,3^ Inflammation in PLWH is multi-factorial and includes immune responses to HIV, opportunistic infections, and microbial products which translocate from the lumen of the gastrointestinal (GI) tract, as well as cytokine-driven bystander inflammation.^3–5^ Understanding the mechanisms that contribute to this inflammation may lead to the development of therapeutics that improve the morbidity/mortality of PLWH.

The contribution of microbial translocation to HIV disease progression is of considerable interest and immunological and physiological perturbations within the GI tract of individuals with progressive HIV/SIV infections have been widely explored.^6–9^ These perturbations include the early and severe depletion of CD4 + T cells in the mucosa, including the preferential loss of Th17 cells, and the inability of macrophages in the lamina propria to effectively phagocytose translocated microbes.^10–13^ Epithelium integrity is also compromised, due to damage from enterocyte apoptosis and tight junction disruption.^10–12^ Mucosal immune hyperactivation and persistent inflammation occurs alongside these, at least in part due to innate immune activation stemming from the presence of increased microbial translocation that follows from the damaged epithelium.^10^

More recently, how the composition of the GI tract microbiome influences HIV and SIV disease progression has been considered.^10,14–18^ Under normal conditions, the GI tract microbiome is composed of a complex community, with over 1000 species of bacteria, and this composition is important for the healthy physiology of the host.^19,20^ Bacteria in the GI microbiome harbor thousands of genes not possessed by the host, enabling them to produce enzymes and metabolites that assist digestion, help immune maturation, and contribute to proper function of the digestive and nervous systems.^21–23^ Diversity of the GI microbiome is essential for these processes to continue, with different phyla contributing different functions to the host. Members of the Bacillota and Bacteroidota phyla commonly hydrolyze complex carbohydrates that humans cannot metabolize, releasing short chain fatty acids that support intestinal health.^21–23^ Several phyla contribute to the recycling of bile salts and can work in concert to synthesize essential vitamins for the host.^24,25^

Several studies have demonstrated altered GI tract microbiome − termed dysbiosis − in PLWH, particularly when HIV disease progresses to advanced states.^26,27^ The dysbiosis observed involves reduced bacterial diversity and increased levels of taxa belonging to the Pseudomonadota phylum, which include facultatively Gram-negative bacteria which contain many pathogenic genera.^28^ Such dysbiosis is less obvious when samples are longitudinally studied from chronically SIV-infected Asian macaques.^29–31^ Moreover, antibiotic-mediated induction of dysbiosis to reduce taxa belonging to the phylum Bacillota and increasing the abundance of taxa belonging to the phylum Pseudomonadota did not accelerate progressive SIV infection in Asian macaques.^14^ Furthermore, this experimentally induced dysbiosis did not increase markers of microbial translocation − an increase that was expected based on previous work that has shown an enrichment for members of the Pseudomonadota phylum among translocated taxa.^14,29^ This highlights that translocation is not merely an extension of dysbiosis. Thus, a full understanding of how individual bacterial taxa contribute to microbial translocation and disease progression is lacking; a deficiency we aim to contribute to correcting.

Here we isolate, identify, and genetically and functionally characterize replication-competent bacteria that had translocated into the liver, mesenteric lymph nodes, or spleen of chronically SIV-infected rhesus macaques (RM). We confirm that microbial translocation does not seem to be stochastic and demonstrate by proteomic profiling that proteins involved in epigenetic modulations are enriched in translocating taxa. We further demonstrate that treatment with the cytosine methyltransferase inhibitor decitabine significantly impairs the growth of these taxa in vitro and reduces their relative abundance in chronically SIV-infected macaques *in vivo*, though not to a significant degree. These data provide mechanistic insight into the process of microbial translocation and explore a novel therapeutic intervention to curb microbial translocation and the associated inflammation.

## Results

### Bacterial translocators

**Fig. 1.**
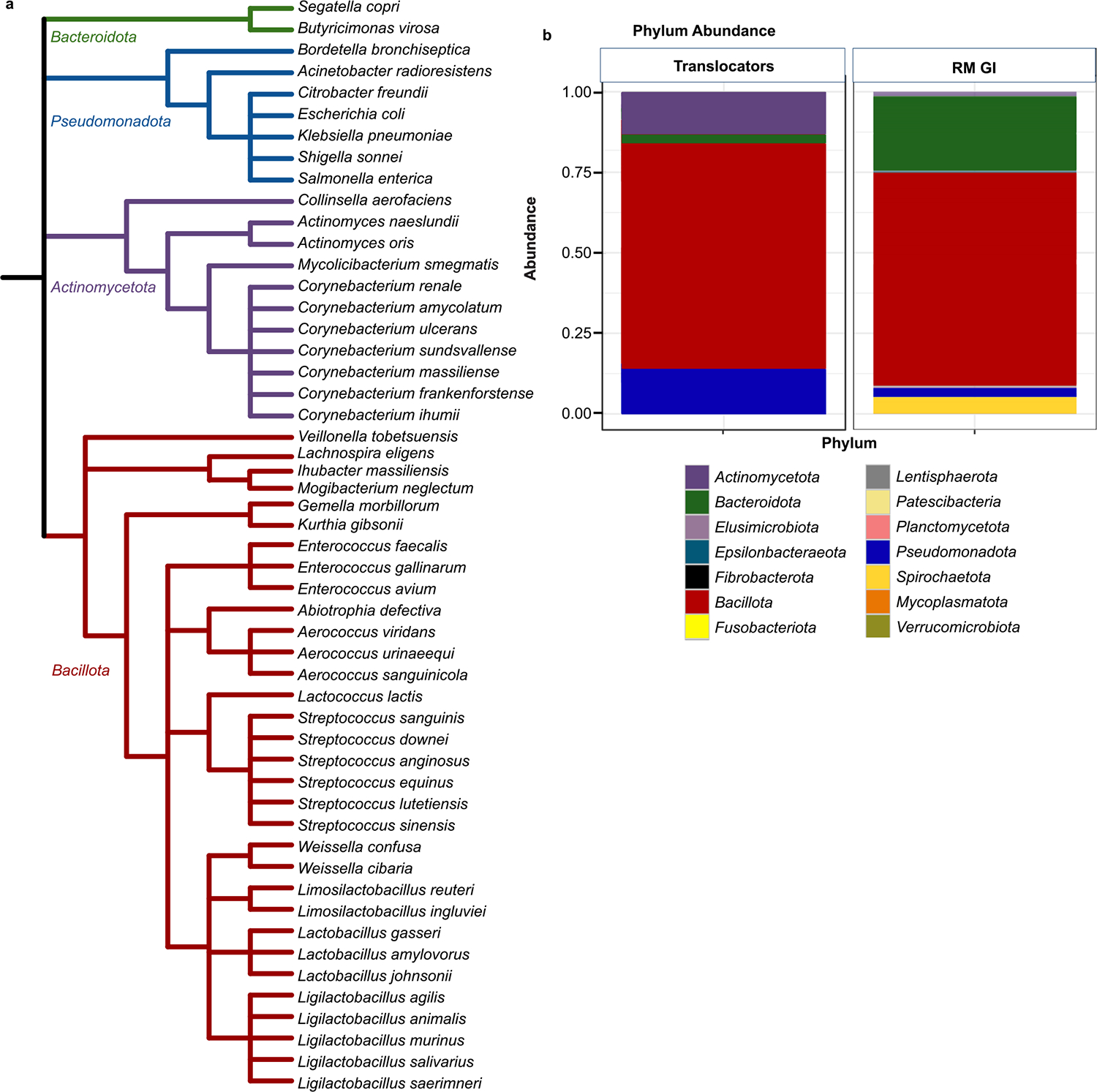
Translocating Species are not representative of the fecal microbiome. a, Phylogenetic tree of the 52 bacterial species isolated from SIV-infected rhesus macaque tissues that were not excluded for further study (n = 22 RM). Branches are color-coded according to phylum: *Bacillota* in red, *Actinomycetota* in purple, *Pseudomonadota* in blue, and *Bacteroidota* in green. b, Phylum relative abundance plot depicting distribution of translocating bacterial isolates compared to the abundance of bacterial phyla typically found in the RM GI microbiome of randomly selected RM housed in our animal facility.

To identify translocating bacterial taxa, liver, mesenteric lymph node, and spleen samples were collected from SIV/SHIV-infected RM (n = 22) that had progressed to AIDS. Samples were homogenized and plated under both aerobic and anaerobic conditions to isolate live bacteria that had translocated from the periphery to these distal sites and individual bacterial taxa were identified by 16S rRNA gene sequencing. 52 different translocated bacterial species were isolated in this manner that passed the exclusion criteria (Fig. 1a). Additional species were isolated but were excluded from further consideration due to high likelihood of being contaminants from the skin microbiome or the environment. While impossible to definitively determine if they were contaminants or genuine translocators from relatively small populations in the gut, we erred on the side of caution by excluding them. When comparing the phylum abundance of the translocating bacterial isolates to a representative RM fecal microbiome composition, and when accounting for the number of times species were isolated from different tissues and animals, clear differences were apparent between the GI tract and distal sites (Fig. 1b). Most bacterial taxa that translocate are believed to do so from the GI tract, hence the comparison of translocating taxa to the composition of the GI microbiome^3,11^. No single bacterial species dominated the total number of isolates, nor was any animal or tissue type a disproportionate source of isolates (Supplementary Fig. 1).

*Bacillota* are among the most represented taxa in the macaque GI tract^31^ as well as among taxa that translocated to distal sites,^29^ with 70% of translocators belonging to the phylum and 65% of bacteria in the GI of our RM being *Bacillota*. As expected, far more *Pseudomonadota* were represented in the distal sites as compared to the fecal microbiome − 14% compared to 2%, respectively. *Actinomycetota* also accounted for a disproportionately higher number of isolates, with 13% of translocators coming from this phylum while only 1% of the GI microbiome in our RM is *Actinomycetota*. *Bacteroidota* however represented a lower-than-expected proportion of translocators at only 3%, compared to the 25% found in the GI of our RM. Thus, microbial translocation does not seem to be stochastic.

### Translocator-specific IgG is not elevated at time of translocation

**Fig. 2.**
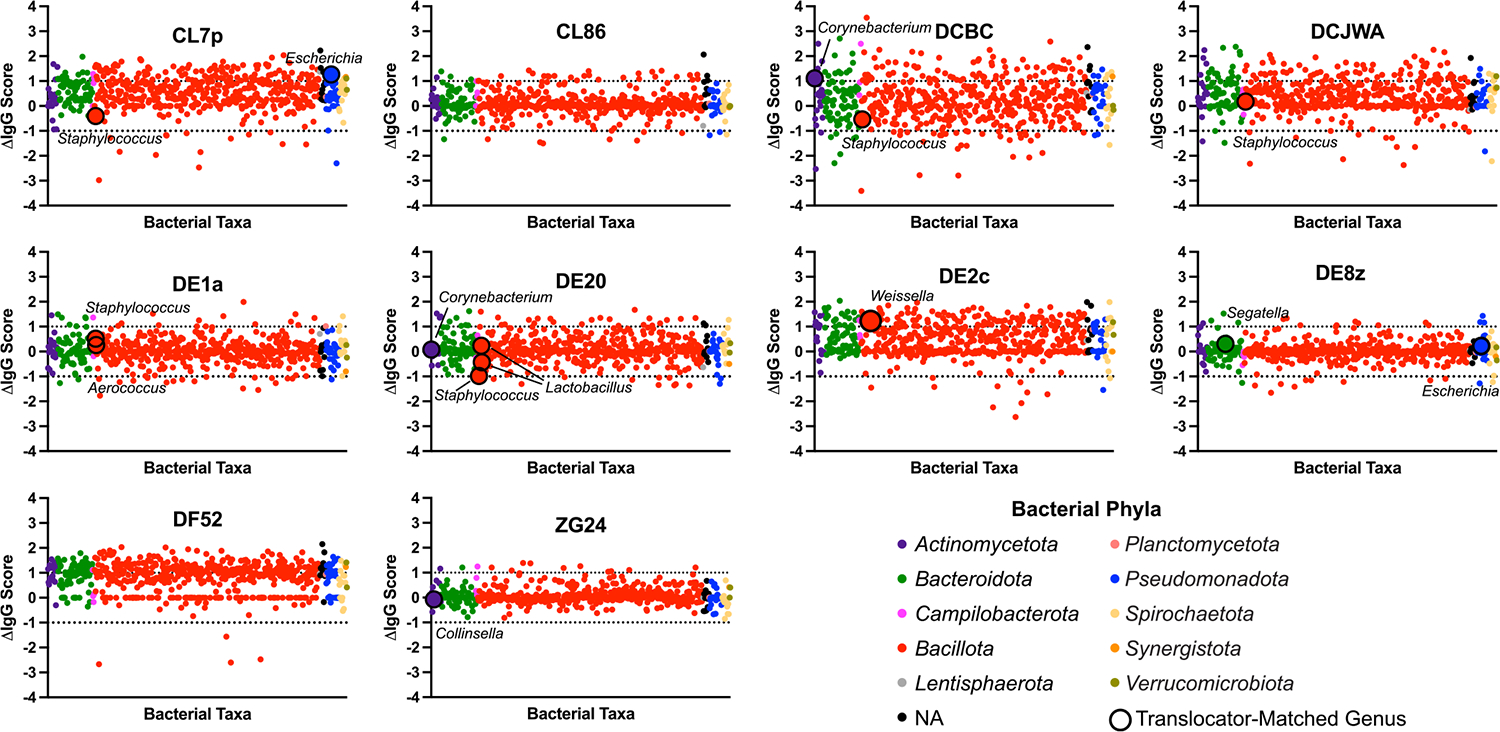
Translocator-Specific IgG is Not Elevated at Time of Translocation. Scatter dot plots of ΔIgG scores from 10 RM. Colored by phylum. Larger dots with black outlines indicate IgG-seq sequences whose genus matched the genus of a translocator bacterial isolate from that animal. Scores below 0 indicate enrichment before SIV-infection while scores above 0 indicate enrichment post-SIV infection, at time of necropsy. Taxa with scores below −1 or above 1 are considered translocators.

Recent data suggest that translocating bacterial taxa induce systemic IgG responses and that IgG-seq can be used to identify translocating taxa.^32^ We performed IgG-seq on 10 paired plasma samples, comparing samples prior to SIV infection and after AIDS progression, from whence translocating taxa were isolated.^32^ IgG scores were calculated as the log_10_ ratio of counts between each taxon in the IgG-enriched microbial fraction over the counts found in the IgG-depleted fraction. Resulting IgG scores for bacterial taxa from the pre-infection time point were subtracted from the IgG scores at time of necropsy to calculate ΔIgG scores (Fig. 2). Per animal, these bacterial taxa were queried for genera matching the genus of any bacteria isolated from the individual animal to determine whether a systemic IgG response was generated in response to identified translocators. Taxonomic level was restricted to genus, rather than species matching, due to resolution of sequencing data from IgG-16S rRNA gene sequencing protocol. Out of 15 genera matches (out of a total possible 41 matches; some translocator genera were not found in the sequencing data), only 3 had a ΔIgG score > 1, which would indicate a greater systemic IgG response to the translocating bacteria during AIDS than during pre-infection. The remaining 12 genera matches had ΔIgG scores > −1 but < 1, indicating no increase in immune response to these genera when RM had progressed to AIDS. These data suggest that changes in IgG against translocating species do not precede or coincide with translocation. This result indicates that these translocating taxa may be poorly immunostimulatory in the context of AIDS, possibly due to immunodeficiency or bacterial function within the host.

### Proteomic analysis of translocators

**Fig. 3.**
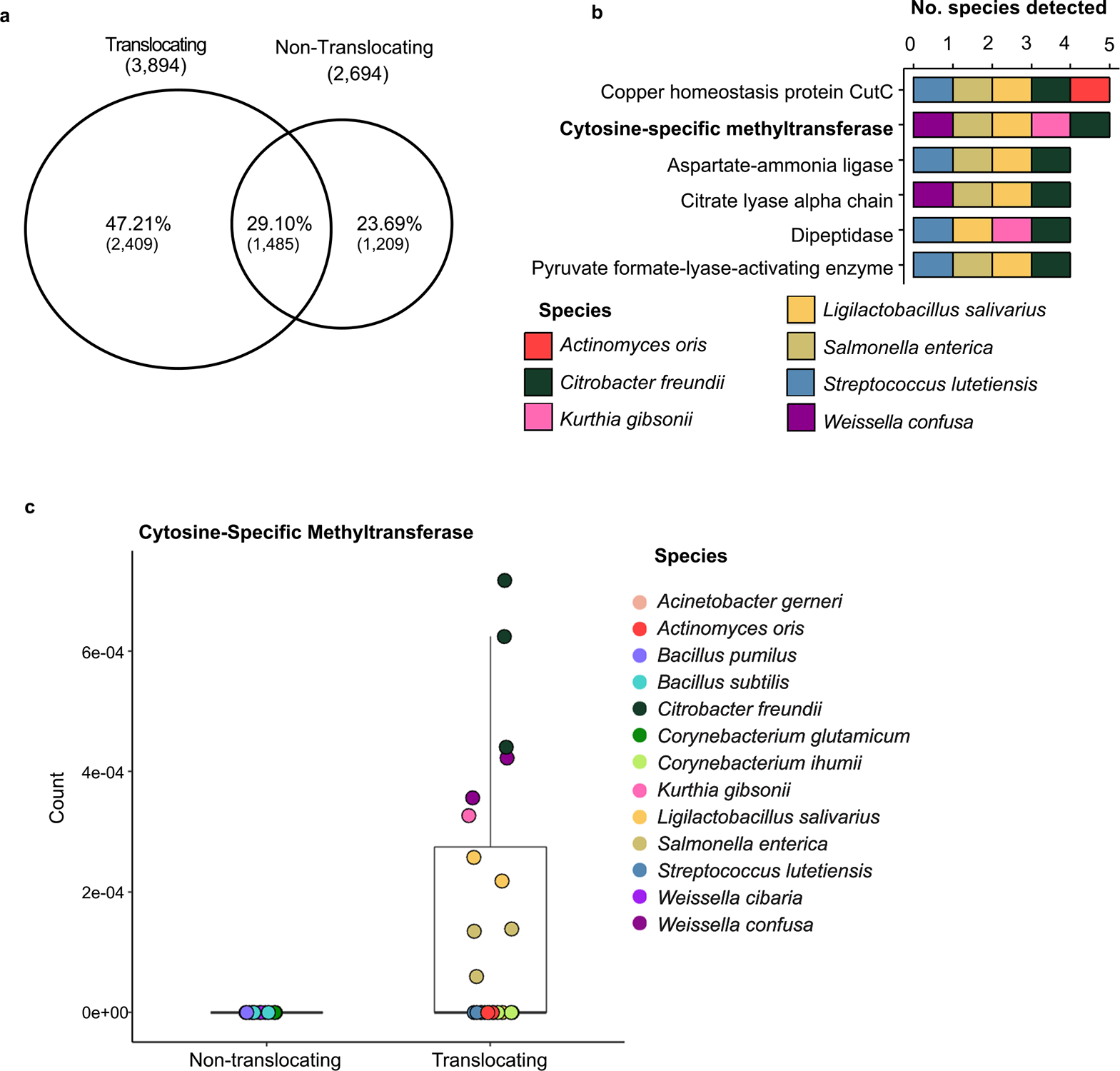
Translocating bacterial taxa uniquely transcribe a cytosine-specific methyltransferase. a, 5,103 proteins were detected across all bacterial samples (n = 13), 2,409 being unique to translocating bacterial taxa. b, 6 proteins were detected in 4 or more translocating bacterial taxa out of 8 tested species. c, Cytosine-specific methyltransferases were detected in 5 out of 8 tested translocating species and were not detected in any non-translocating species. Boxes represent the inter-quartile range.

We next sought to determine if there were common proteins expressed among translocating taxa compared to taxa that did not translocate. We grew 13 bacterial taxa samples − 8 translocating taxa and 5 non-translocating taxa − and used mass spectrometry to study their proteomes, with three technical replicates per sample being analyzed. Across all 13 taxa 5,103 unique proteins were detected by mass spectrometry. (Fig. 3a). Of these, 2,409 were unique to the translocating taxa. Among these 2,409 proteins, 6 were found in 4 or more of the translocating bacterial species (Fig. 3b). One of only two proteins to be detected in 5 out of 8 translocating taxa was a cytosine-specific methyltransferase. Cytosine methylation is relatively uncommon in bacteria^33^ and this protein was not detected in any non-translocating species; for these reasons we chose to explore the role of this protein in more depth (Fig. 3c). These data are consistent with the premise that microbial translocation is not stochastic and that specific genes may facilitate translocation.

### In vitro bacterial responses to decitabine

**Fig. 4.**
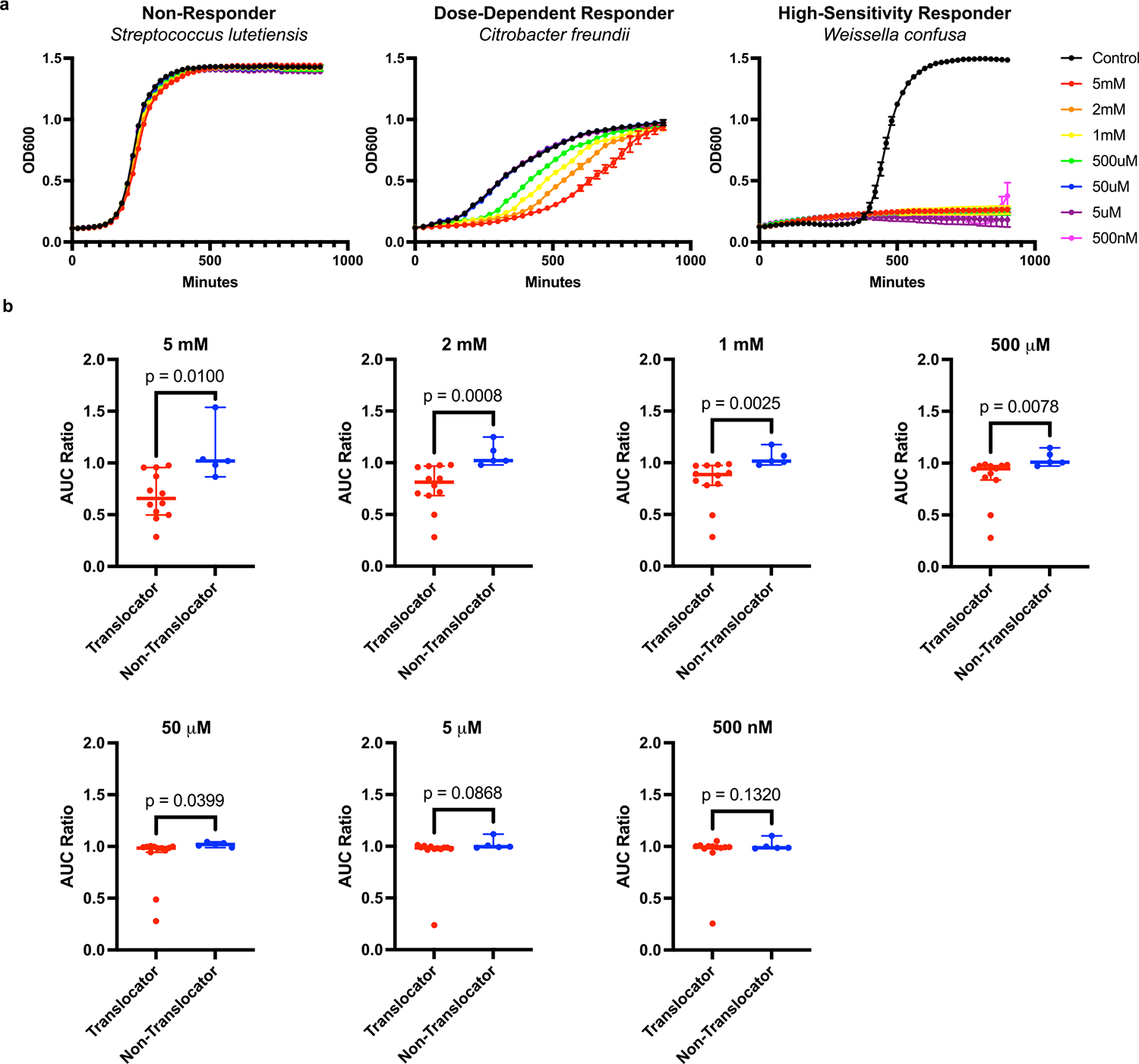
*In Vitro* Decitabine Growth Assays Reveal Greater Inhibition of Translocators. a, Representative growth curves of bacteria grown in the presence of decitabine across a range of concentrations. From left to right, non-responders, dose-dependent responders, and high-sensitivity responders. Bars above and below represent the standard deviation of the data for samples run in triplicate. b, Area Under the Curve ratio comparisons between translocating and non-translocating bacterial taxa for each concentration of decitabine tested (n = 12 translocating, n = 5 non-translocating). Significant differences between groups determined by one-tailed Welch’s *t*-test. Bars represent median with 95 % CI.

To understand if DNA methylation is an important biological function for translocating taxa, a subset of translocating bacterial taxa were grown in the presence of decitabine, a hypomethylating agent that inhibits the activity of cytosine-specific methyltransferases.^34^ Growth kinetics were measured by optical density in the presence of decitabine in concentrations from 5 mM to 500 nM (Fig. 4). Representative examples of typical response types to decitabine treatment over 15 h are shown in Fig. 4a: non-responders, dose-dependent responders, and high-sensitivity responders demonstrate decreased growth capacity by translocating taxa. Growth curves for all assayed species are shown in Supplementary Fig. 2. Area under the growth curve ratios were compared between translocators (n = 13) and non-translocators (n = 5) for their responses to each of the tested concentrations of decitabine (Fig. 4b). Translocator growth was significantly more inhibited by the presence of decitabine at concentrations of 5 mM (p = 0.0100), 2 mM (p = 0.0008), 1 mM (p = 0.0025), 500 μM (p = 0.0078), and 50 μM (p = 0.0399). Differences in growth inhibition between translocators and non-translocators were not significant at decitabine concentrations of 5 μM (p = 0.0868) and 500 nM (p = 0.1320). These data demonstrate that expression of cytosine methyltransferases is important for the growth of translocating bacterial taxa and may represent a druggable target to specifically reduce microbial translocation.

**Fig. 5.**
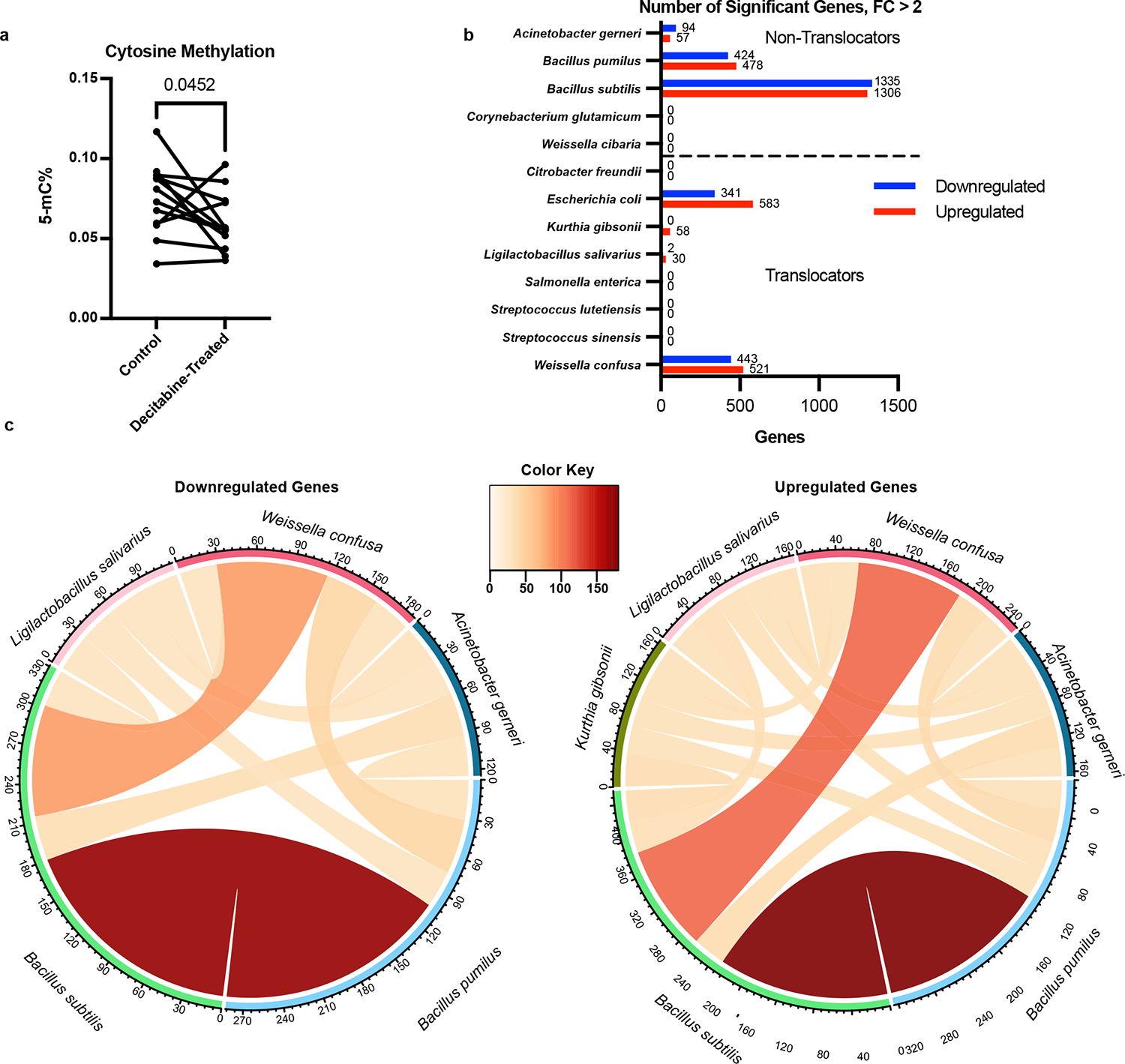
Molecular Responses to Decitabine Indicate Bacteriostatic Effect in Translocators. a, 5-mC% of genomic DNA isolated from translocating and non-translocating bacterial species (n = 12) incubated for 6 h with or without decitabine. Significant difference determined by one-tailed paired *t*-test. b, Number of significantly differentially expressed genes detected in total-RNAseq for each bacterial species tested (n = 13, 5 non-translocators and 8 translocators), after 6 h of growth with or without decitabine. Significant changes determined using the Wald test in DESeq2. c, Circos plots depicting the number of overlapping downregulated or upregulated orthologous gene clusters, as determined by OrthoVenn2, shared between species that had significant changes in gene expression with decitabine treatment.

To explore potential mechanisms responsible for the growth inhibition mediated by decitabine, bacterial taxa (n = 12 [8 translocators, 4 non-translocators]) were grown in the presence or absence of decitabine for 6 h and then assessed for global DNA 5-methylcytosine% (5-mC%) using ELISA^35^ (Fig. 5a). Decitabine-treated bacteria had significantly lower global 5-mC% compared to the untreated controls (paired *t*-test, p = 0.0452), confirming the efficacy of decitabine in our system. To determine if there was a common downstream response to decitabine treatment in how translocators (n = 8) and non-translocators (n = 5) responded to decitabine treatment, bacteria were cultured for 6 h with or without decitabine and assessed for changes in their transcriptomes using bacterial RNAseq (Fig. 5b). We grew bacteria in the presence or absence of decitabine (concentration determined by response in growth assays), and isolated RNA for RNASeq. DESeq2 analysis of identified genes demonstrated that several taxa had differentially expressed genes in response to treatment. 3 out of 5 non-translocating species had significantly downregulated and upregulated genes (p < 0.1). Meanwhile, only 2 out of 8 translocating species had significantly downregulated transcripts and 4 out of 8 had significantly upregulated genes. Protein sequences extracted from differentially expressed genes were used to identify orthologs and compare differentially abundant genes between bacterial species using OrthoVenn2^36^ (Fig. 5c). Surprisingly, no broadly common response to decitabine appears to exist between species with significant differential gene expression after treatment. These data, coupled with the results of the growth assays with decitabine, suggest that decitabine treatment stalled bacterial growth and was bacteriostatic.

### In vitro bacterial DNA stimulation of PBMCs

Unmethylated CpG motifs, commonly found in bacterial DNA, are immunostimulatory to the immune system − particularly B cells − via Toll-like receptor (TLR) 9^37^. To test if the presence of cytosine-specific methyltransferases among translocating bacterial taxa would reduce these immunostimulatory effects, bacterial DNA from translocators and non-translocators was used to stimulate peripheral blood mononuclear cells (PBMC) from SIV-uninfected (n = 4) and SIV-infected (n = 2) RM (Supplementary Fig. 3).We used flow cytometric analysis to measure CD69, HLA-DR, and Ki67 in B cells (gating strategy shown in Supplementary Fig. 3a). While IgM+B cells showed a significant increase in side-scatter when stimulated with translocator DNA compared to non-translocator DNA (indicative of B cell blasting, unpaired *t* test, p = 0.0018), no markers of activation or blasting were significant after correcting for multiple comparisons (Supplementary Fig. 3b). These data suggest that cytosine methyltransferase expression does not reduce the capacity of the bacterial DNA to stimulate through TLR9, and immune evasion may not underscore the mechanism responsible for preferential translocation of particular bacterial taxa.

### Decitabine does not improve markers of microbial translocation in SIV-Infected RM

**Fig. 6.**
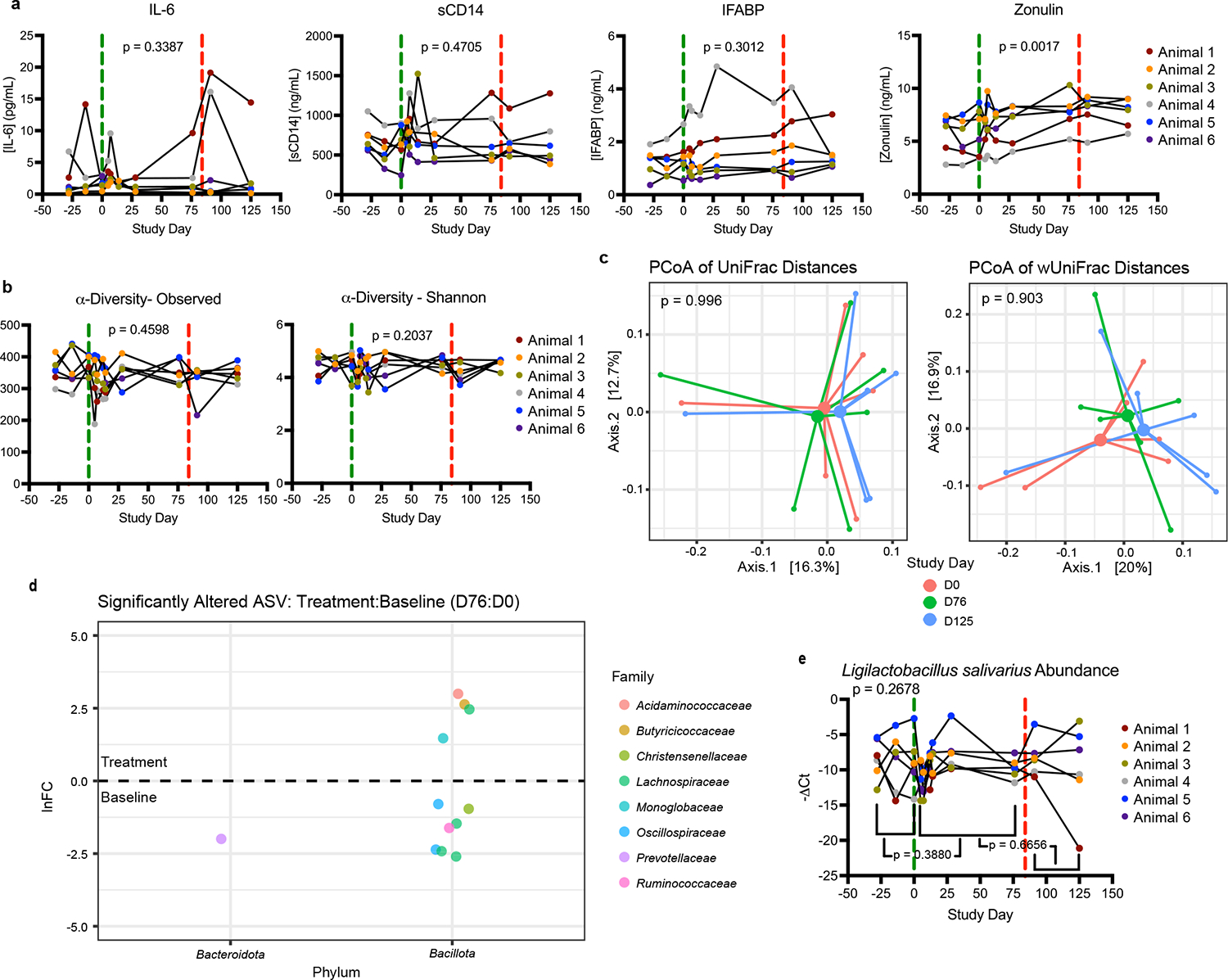
*In Vivo* Decitabine Treatment Induces Mild Changes in Host and GI Microbiome. a, Concentrations in plasma of IL-6, sCD14, IFABP2, and zonulin from decitabine-treated SIV-infected RM (n = 6). Significant changes determined by one-way ANOVA or mixed-effects model. The vertical dashed green line indicates when treatment began and the vertical dashed red line indicates when treatment was stopped. b, Observed and Shannon α-diversity of the GI microbiome from decitabine-treated SIV-infected RM (n = 6). Significance across study determined by one-way ANOVA. The vertical dashed green line indicates when treatment began and the vertical dashed red line indicates when treatment was stopped. c, Principal coordinate analysis of unweighted and weighted UniFrac distances (β-diversity) of decitabine-treated SIV-infected RM (n = 6), between baseline (D0), treatment (D76), and washout (D125) phases of the study. Significant changes between time points assessed by Adonis. Lines represent the distance of each sample to the group’s centroid. d, Ln fold changes of significantly altered ASVs between baseline and treatment, as determined by ANCOM-BC (q-value < 0.05). e, Changes in the relative abundance of *Ligilactobacillus salivarius* in the stool of decitabine-treated SIV-infected RM (n = 6). Significance over the course of treatment determined by one-way ANOVA, with multiple comparisons of means used to determine significant differences between individual time points. The vertical dashed green line indicates when treatment began and the vertical dashed red line indicates when treatment was stopped.

Decitabine is a chemotherapeutic agent used to treat acute myeloid leukemia and myelodysplastic syndromes. To understand if this compound might be repurposed to reduce microbial translocation, we treated 6 chronically SIV-infected RM 3 times per week for 12 weeks with oral 1.25 mg/kg of decitabine and measured markers of inflammation and microbial translocation, longitudinally. While normally administered intravenously or subcutaneously^38^, decitabine was administered orally to more specifically target its effects against the contents of the GI lumen − the primary source of microbial translocators – in an effort to stop microbial translocation before it could occur. IL-6 and sCD14 were quantified from plasma to monitor immune activation and microbial translocation, respectively (Fig. 6a). Neither biomarker significantly changed over the course of treatment (one-way ANOVA, p = 0.3387 and p = 0.4705, respectively). Plasma levels of intestinal fatty acid binding protein (IFABP) and zonulin were also quantified, to assess damage to the GI tract during the study period (Fig. 6a). IFABP did not significantly change over the course of treatment (one-way ANOVA, p = 0.3012), but zonulin significantly increased with treatment and into the washout phase of the study (one-way ANOVA, p = 0.0017). B cells, T cells, monocytes/macrophages, and NK cells from biopsies from the jejunum, liver, and rectum were assessed by flow cytometry for immune cell phenotypes and functionality, but no significant differences were seen between baseline and treatment time points (Supplementary Fig. 4a). Similarly, there were no significant changes in viral load or liver enzymes in the blood over the course of the study (Supplementary Fig. 4b). These data suggest decitabine was not sufficient to reduce inflammation and may have caused damage to the GI tract epithelial barrier.

### Decitabine Induces Mild changes in the GI microbiome

To understand if oral decitabine treatment altered the composition of GI bacterial communities, we characterized the fecal microbiome by 16S rRNA gene Illumina sequencing. Among identified amplicon sequence variants (ASVs), neither observed nor Shannon α-diversity measures significantly changed with decitabine treatment (Fig. 6b) (one-way ANOVA, p = 0.4598 and p = 0.2037, respectively). Unweighted and weighted UniFrac distances, used to assess β-diversity, also did not significantly change between days 0 (pre-treatment), 76 (11 weeks of treatment), and 125 (6 weeks post-treatment) (Fig. 6c) (Adonis, p = 0.996 and p = 0.903, respectively). Analysis of Compositions of Microbiomes with Bias Correction (ANCOM-BC) was used to assess the sequencing data for significantly altered ASVs between days 76 and 0 of the study (Fig. 6d). Two phyla showed significantly altered ASVs: 1 taxon from *Bacteroidota* and 11 from *Bacillota*. The 16S rRNA gene sequencing taxonomic assignment depth was limited to genus level, so qPCR was used to determine the relative fecal abundance of the most commonly isolated translocating species, *Ligilactobacillus salivarius* (Fig. 6e). Overall there was not a significant change in its relative abundance (one-way ANOVA, p = 0.2678), with comparisons between treatment periods (Pre-Treatment vs Treatment and Treatment vs Post-Treatment) showing non-significant changes while the RM hosts were receiving decitabine treatment (p = 0.3880 and p = 0.6656, respectively). These data are consistent with decitabine being bacteriostatic and may reduce translocating bacterial taxa from the GI tract microbiome.

## Discussion

Chronic inflammation persists as an issue for PLWH despite antiretroviral treatment and is driven, in part, by microbial translocation – the dissemination of bacteria from the GI lumen into systemic circulation. Identifying features that are unique to these bacterial translocators is an important first step in developing therapies to mitigate microbial translocation and the downstream inflammation to which it contributes. Here we studied potential bacterial-specific mechanisms of microbial translocation by isolating, identifying, and analyzing the proteomics and transcriptomics of bacterial taxa isolated from non-barrier sites in chronically SIV-infected rhesus macaques. We found that an enrichment of a previously uncharacterized cytosine-specific methyltransferases was common among cultured, translocated bacterial isolates. These methyltransferases stood out for further investigation for two reasons: cytosine methylation is uncommon relative to adenosine methylation in bacteria^33^ and they were not detected in any of the tested non-translocating taxa. Compared to non-translocating taxa, we found that the growth of translocating bacterial taxa was significantly more inhibited by decitabine, an inhibitor of cytosine-specific methyltransferases. We also found a higher proportion of translocators than non-translocators to have little to no differentially expressed genes in response to decitabine treatment, suggesting that decitabine is likely acting as a bacteriostatic agent rather than a bactericidal one. This, combined with a lack of finding any restriction enzymes to be enriched in the proteomic results, may indicate that the methyltransferases enriched in translocators are orphan methyltransferases rather than a part of a Type II restriction-modification system meant to protect against bacteriophage. Orphan methyltransferases are commonly regulators of cell cycle processes, but in many species are not essential for survival;^39^ within this context, decitabine acting as a bacteriostatic agent is fitting.

Previous studies have shown that translocating bacterial taxa can exhibit enhanced immune evasion,^40^ a characteristic that could be present in the translocators isolated in our study and mediated by the methylation of bacterial DNA itself. With our translocators being enriched for cytosine-specific methyltransferases, we reasoned that their DNA may be less immunostimulatory than the DNA from non-translocators. Indeed, toll-like receptor 9 (TLR9) activation stimulates immune cells upon the recognition of unmethylated CpG motifs.^41^ However, we found no significant differences in activation markers of B-cells and T-cells stimulated with DNA isolated from bacteria in our study. How, or if, translocating bacteria evade humoral and cellular immunity would be important to study.

*In vitro* studies are inherently limited, with single bacterial species cultures failing to capture the complexity of the microbiome within the gastrointestinal tract of an animal. To more globally assess the contribution of cytosine-specific methyltransferases on microbial translocation in the context of lentiviral infection, we assessed the effects of decitabine treatment on host markers of microbial translocation and the host GI microbiome in chronically SIV-infected rhesus macaques. Immune cells in the blood, liver, jejunum, and rectum showed no changes in response to treatment, indicating no adverse effects of treatment on host immune cell populations. Decitabine treatment also had no effect on systemic immune activation or microbial translocation as evidenced by unperturbed IL-6 and sCD14 plasma levels over the course of the study. Plasma markers of intestinal immune activation yielded mixed findings, however. Whereas IFABP plasma levels did not change with treatment, plasma zonulin levels increased with treatment, indicating that decitabine may have made intestinal damage worse. This may be due to off-target effects on the rapidly dividing epithelial cells that line the GI tract – indeed, the hypomethylating effects of decitabine that make it effective in treating cancers by reactivating silenced tumor suppressor genes could very well cause deleterious epigenetic changes in healthy cells.^34^ Further experiments are needed to explore these findings. A detailed assessment of the fecal microbiome revealed that although there were significant differences in a number of ASVs, α-Diversity and β-diversity of the GI microbiome did not significantly change with decitabine treatment. These results together indicate that decitabine can alter the abundance of relatively few taxa without causing large scale alterations in the GI microbiome like those commonly seen with antibiotic treatments. This is likely due to the relative infrequency of cytosine methylation across the bacterial kingdom. Although few of the perturbed taxa match with isolated translocators at the genus level, these results may not represent the concentration of taxa interfacing with the epithelial barrier. Overall, the results of our *in vivo* study indicate that our course of decitabine was insufficient to inhibit microbial translocation and may have contributed to worsening intestinal damage at the concentration and duration used in our study.

The selective targeting of potential translocators has the potential to remove a source of chronic inflammation, as opposed to individually treating the multitude of co-morbidities resulting from inflammation. Because decitabine was undetectable in stool supernatants, non-efficacy in our study – an inability to reduce systemic markers of microbial translocation- may be due to an insufficient concentration of decitabine in the gut lumen. In addition to potential adjustments in dose and dosing frequency, alternative hypomethylating agents could be tested, including the orally bioavailable prodrug of decitabine^42^ OR-2100, or, alternative delivery methods such as hydrogels or nanoparticles that could more selectively deliver therapeutics to the epithelial barrier.

In summary, our results show that bacterial translocators within the context of SIV infection are enriched for cytosine-specific methyltransferases, identifying a novel therapeutic target to alleviate chronic inflammation induced by translocation. How these findings relate to other diseases where microbial translocation occurs merits investigation.^43^ Understanding bacterial-mediated mechanisms which contribute to preferential microbial translocation may enable improved therapeutics to manage non-AIDS defining comorbidities for the millions of people living with HIV.

## Online methods

### Bacterial isolation/identification

The 22 rhesus macaques (RM) (*Macaca mulatta*) used for isolation of translocating bacteria were previously infected with SIV/SHIV and allowed to progress to clinical AIDS (loss of > 25 % body weight from baseline weight; opportunistic infection; major organ failure or medical conditions unresponsive to treatment) before tissue sampling (Supplementary Table 1). The NIAID Institutional Animal Care and Use Committee, as part of the NIH intramural research program, approved all experimental procedures pertaining to NHPs (protocol LVD 26E). The animals in this study were housed and cared for under the supervision of the Association for the Assessment and Accreditation of Laboratory Animal Care (AAALAC)-accredited Division of Veterinary Resources and as recommended by the Office of Animal Care and Use nonhuman primate management plan. Care at these facilities met the standards set forth by the Animal Welfare Act, animal welfare regulations, United States Fish and Wildlife Services regulations, as well as the 8th edition of the Guide for the Care and Use of Laboratory Animals.^44^ The physical conditions of the animals were monitored daily. Animals in this study were exempt from contact social housing due to scientific justification, per Institutional Animal Care and Use Committee protocol, and were housed in non-contact social housing where primary enclosures consisted of stainless-steel primate caging. Animals were provided continuous access to water and offered commercial monkey biscuits twice daily as well as fresh produce, eggs, and bread products twice weekly and a foraging mix of raisins, nuts, and rice thrice weekly. Enrichment to stimulate foraging and play activity was provided in the form of food puzzles, toys, cage furniture, and mirrors or television.

When animals had progressed to AIDS, euthanasia was initiated using protocols consistent with the American Veterinary Medical Association guidelines. Animals were sedated with Telazol at 4 mg/kg i.m., followed by Pentobarbital at 80 mg/kg to reach euthanasia. Tissues were maintained in RPMI or Anaerobic Transport Media (Anaerobe Systems) before processing. Liver, spleen, and mesenteric lymph nodes were homogenized in PBS using a gentleMACS dissociator (Miltenyi) using the RNA_01 program for aerobically-processed samples or by passing through a 0.22 μm cell strainer for anaerobically-processed samples. 100 μL of homogenate was plated on: a) Brain Heart Infusion, b) Tryptic Soy Agar (TSA) + Tween 80, and c) TSA+5% Sheep’s Blood media (Teknova) under aerobic conditions, and d) Brucella Blood and e) CDC Blood media (Anaerobe Systems) under anaerobic conditions. Plates were incubated for 1–7 days at 37 °C to achieve colony density, with colonies re-streaked on fresh plates for purity.

Pure cultures on plates were used to inoculate liquid media of the same type that bacteria were isolated on. DNA was extracted from these liquid cultures using the Qiagen DNeasy Blood and Tissue kit per manufacturer protocol, with all samples pretreated as if gram-positive. Purified DNA was PCR amplified for the 16S rRNA gene (27f 5′-AGAGTTTGATCMTGGCTCAG-3′, 1492r 5′-GGTTACCTTGTTACGACTT-3′), then for sequencing (Eurofins). Isolate identities were assigned by running sequences through the NCBI nucleotide BLAST database.

### IgG-seq

We performed IgG-seq on paired plasma samples from before infection compared to necropsy timepoint for 10 out of 22 animals (based on availability of samples) that were used in bacterial translocator isolation. Using pooled RM stool samples, IgG-seq was performed as previously reported^32^. Pooled samples were used to ensure capture of IgG titers against the greatest number of RM fecal microbiota members. 500 mg of stool from each of 15 RM was combined as previously described^32^ to generate the RM stool pool. IgG scores from pre-infection were subtracted from the IgG score at the necropsy timepoint to calculate the ΔIgG score for each ASV identified in IgG-seq. Taxa with ΔIgG scores > 1 are considered to be enriched in the necropsy period, and < −1 are considered enriched in the pre-infection period. As ASV taxonomy assignment was reliable only down to genus level using the SILVA taxonomic framework, the genera of translocators isolated from individual animals were searched for within the ASVs of the matching animal’s IgG-seq dataset. Matched genera are indicated on the graphs in Fig. 2 by increased size and outlining of the corresponding point in black.

### Proteomic profiling

Bacteria (n = 8 translocators, n = 5 non-translocators) were grown overnight in MRS media (Teknova) and centrifuged at 5,000 xg for 10 min into pellets of 10^7^ cells (based on OD_600_ estimates), then frozen at −80 °C before further processing. Species were selected based on culturability and to provide as high a representation of the isolated bacterial phyla as possible. Bacterial pellets were prepared for mass spectrometry as previously described^45,46^. Briefly, pellets were resuspended in 350 μL lysis buffer (2 % SDS, 0.1 M dithiothreitol, 0.5 M HEPES), with 50 μL 0.1 mm glass beads then added. Samples were vortexed, heated for 5 min at 95 °C, then vortexed 3 min. Samples were then centrifuged for 3 min at 3000 rpm to pellet beads and cells and the supernatant was transferred to a fresh tube on ice; this process was repeated 3 times. The 2-D Quant assay (GE Healthcare) was used to quantify proteins, with samples then denatured with 8 M urea, alkylated using 50 mM iodoacetamide, treated with benzonase solution (250 U/μL benzonase, 50 mM MgCl_2_, 50 mM HEPES), then digested to peptides using trypsin. Reverse-phase liquid chromatography (LC) was used to clean peptides of salts and detergents using the step-function gradient. Peptides were quantified using a LavaPep Fluorescent Peptide and Protein Quantification Kit (Gel Company) following the manufacturer’s protocol. 1μg of peptide per sample was re-suspended in 2 % acetonitrile, 0.1 % formic acid; this was submitted for nanoflow-liquid chromatography-tandem mass spectrometry (LC-MS/MS) analysis.

Peptide samples were loaded onto a C18-reversed phase Easy Spray column (Thermo Fisher Scientific) and separated on the same column. Peptides were eluted with a linear gradient of 5–22 % buffer B (98 % acetonitrile, 0.1 % formic acid) over 100 min, 22–32 % buffer B for 15 min, 32–90 % buffer B for 5 min, and washed with 90 % buffer B for 10 min at a constant flow rate of 200 nl/min. Each sample was separately analyzed with a nano-flow Easy nLC 1000 connected in-line to a Q-Exactive Plus mass spectrometer with a nanoelectrospray ion source at 2 kV (Thermo Fisher Scientific). Total LC-MS/MS run-time was approximately 180 min, including loading, linear gradient, column wash, and equilibration.

Protein database searches were conducted against an in-house metaproteome database (Logan) using Mascot (v.2.4.0, Matrix Science) and included human proteins from the SwissProt/UniprotKB database to limit possible homologous identifications. Results were imported into Scaffold software to validate these protein identifications, then filtered using the following criteria: <0.1 % FDR for peptide identification, ≤1% FDR for protein identification, and at least two unique peptides identified per protein. Protein spectral counts were normalized to total protein detected.

### Decitabine growth assays

Bacteria were grown overnight in MRS media (Teknova) to revive from glycerol stocks (n = 12 translocating, n = 5 non-translocating). MRS media was prepared to yield the following final concentrations of decitabine (APIChem): 5 mM, 2 mM, 1 mM, 500 μM, 50 μM, 5 μM, and 500 nM. 190 μL of this media was transferred to a 96-well plate in triplicate, along with base MRS media. 10 μL of bacterial overnight culture was used to inoculate each well. Plates were sealed with optical adhesive and cultured for 15 h in a BioTek Epoch2 microplate reader at 37 °C with 200 rpm shaking. OD_600_ readings were taken every 10 min. Data was plotted and area under the curve (AUC) calculated for each condition using Prism v9.3.1. The AUC of each decitabine concentration was divided by the AUC of the base MRS media to determine inhibitory effect of decitabine on growth (AUC ratio < 1 indicating an inhibitory effect of decitabine).

### CpG methylation

Bacteria were grown overnight in MRS media to revive from glycerol stocks (n = 12). 0.2 mL of overnight broth was added to 50 mL MRS media (Teknova) or 50 mL MRS media containing decitabine (concentration determined by sensitivity of species in *in vitro* growth assays), then incubated for 6 h at 37 °C with 150 rpm shaking. DNA was then extracted using the Genomic-tip 500/G (Qiagen) DNA extraction kit according to manufacturer protocol. Extracted DNA was then used in the MethylFlash^™^ Global DNA Methylation (5-mC) ELISA Easy Kit (EpigenTek) and 5-mC% calculated per manufacturer protocol.

### Total RNA-seq

Bacteria were grown overnight in MRS media (Teknova) to revive from glycerol stocks. 0.2 mL of overnight broth was added to 50 mL MRS media or 50 mL MRS media containing decitabine (concentration determined by sensitivity of species in *in vitro* growth assays), then incubated for 6 h at 37 °C with 150 rpm shaking. RNA was then extracted using the RNeasy Mini Kit (Qiagen) per manufacturer’s instructions. Sample libraries were constructed using the Illumina^®^ TruSeq^®^ Stranded Total RNA Sample Preparation kit. Libraries were then sequenced by NovaSeq 6000, SP 200 Cycle (v1.5).

The reference for *Escherichia coli* O157:H7 str. Sakai plasmid pO157 was downloaded from NCBI (GCF_000008865.2).^47^ The reference genomes for the remaining 12 strains were sequenced and assembled by CD Genomics. Sequencing was done through the Illumina HiSeq PE150 platform. Quality control and adapter trimming was performed with bcl2fastq and porechop for Illumina and ONT sequencing respectively. Hybrid assembly with Illumina and ONT reads was performed with Unicycler.^48^ Assembly annotation was performed with Prokka.^49^ The genome sequences were converted from GenBank format to Fasta files using SeqIO from Biopython^50^.

The RNA-seek v1.7.0 workflow was used for processing of the sequencing data. Custom reference genomes were built for all strains using the “build” function in the RNA-seek pipeline. Initial data pre-processing included read quality check with FASTQC v0.11.9, screening for sources of contamination (FQScreen v0.13.0, Kraken v2.1.1), removal of adapters and short sequences, and quality trimming with Cutadapt v1.18. Pre-processed reads were then mapped to the respective reference genomes using STAR v2.7.6a aligner. The aligned bam files were further mapped to the respective genomes using the multimapping option (−M) in featureCounts from Subread v2.0.3. Differential gene expression was performed using DESeq2 v1.36.0. Absolute fold change > 2 and p-value < 0.1 were considered significant. Fold change threshold was relaxed to 1.5 in cases where the higher fold change resulted in no significant genes. Protein sequences were extracted for genes with significant expression using GenomicFeatures v1.50.4, Biostrings v2.66.0, and Rsamtools v2.14.0 in R version 4.2.1. These protein sequences were then analyzed by Orthovenn2,^36^ with sequences annotated using the non-redundant protein database in UniProt and then sorted into clusters of orthologous proteins to identify similarities in the significant genes across species. *Escherichia coli* was excluded from the OrthoVenn2 analysis due to the different genome reference source causing a large skew in the orthologs results. Gplots v3.1.3, ggplot2 v3.4.1, and circlize v0.4.15 were used for graphical representation of the RNA sequencing data and OrthoVenn2 results.

### Animal intervention and sampling

Six SIV-infected RMs (2 female and 4 male), aged 6–16, were treated orally with decitabine (APIChem, 1.25 mg/kg) 3 times a week for 12 weeks by placing the drug inside a food item. Blood, stool, and jejunal, liver, and rectal biopsies were collected longitudinally starting 4 weeks prior to decitabine treatment − allowing each animal to act as its own control in the study − and continuing for 6 weeks after treatment was stopped. Animals were sedated with Ketamine HCl at 10 mg/kg intramuscular (i.m.) for longitudinal blood and stool sampling or with Telazol at 3–4 mg/kg i.m. for tissue timepoints. For rectal biopsies, animals were further anesthetized with isoflurane gas by intubation to effect. Successful anesthetization was monitored by response to stimuli. No animals met endpoint criteria as defined by: (a) loss of 25 % body weight from baseline weight when assigned to protocol, (b) major organ failure or medical conditions unresponsive to treatment, (c) complete anorexia for 4 days or an inability to feed or drink sufficient nutrients to maintain body weight without assistance for 7 days, (d) distress vocalization unresponsive to treatment or intervention for 7 days, or (e) tumors arising from other than experimental means that grew in excess of 10 % of body weight, impaired movement, or ulcerated.

Whole blood was collected into EDTA. For longitudinal intestinal biopsies, fecal material was removed from the rectum and biopsies obtained with biopsy forceps. 10 pinch biopsies were obtained per animal for longitudinal assessments. For liver biopsies a small section of tissue was removed via laparoscopy, approximately 1 cm being cut from the end of a lobe. Biopsies were transported in RPMI and washed twice in PBS prior to processing. Approximately 1 mL of RM stool was collected fresh from each animal by inserting a sterile swab 2 cm into the rectum and spinning to collect available sample. Collected feces were snapfrozen and stored at − 80 °C until DNA isolation.

Plasma was isolated from whole blood by centrifugation. Mononuclear cells were isolated from blood by Ficoll gradient centrifugation and from biopsies by straining/grinding samples through a 0.22 μm cell strainer.

### Plasma biomarker quantification

Concentrations of IL-6 (R&D HS600C), sCD14 9R&D DC140), IFABP (MyBioSource MBS740424), and Zonulin (Alpco 30-ZONSHU-E01) were quantified from plasma using commercially available ELISA kits, performed according to the manufacturer’s protocols.^51^ Plasma viral RNA levels were determined by qRT-PCR as previously described.^52^

### Immune phenotyping and functional assessment

Polychromatic flow cytometry was done using a Cytek^®^ Aurora (SpectroFlo^®^ v3.0.3). Antibodies against the listed antigens were used for staining at predetermined concentrations: CCR4 (clone 1G1), CCR5 (3A9), CCR7 (3D12), CD11b (ICRF44), CD14 (M5E2), CD20 (2H7), CD28 (CD28.2), CD3 (SP34–2), CD4 (SK3), CD45 (D058–1283), FoxP3 (259D/C7), HLA-DR (G46–6), IFN-g (B27), IgG (G18–145), IgM (G20–127), Ki67 (B56), and TNF-α (MAb11) from BD; CD16 (3G8), CD8 (RPA-T8), CD95 (DX2), IL-17a (BL168), IL-2 (MQ1–17H12), and PD-1 (EH12.2H7) from Biolegend; CD159a/NKG2a (Z199) from Beckman; CD154/CD40L (24–31) and IL-22 (IL22JOP) from eBioscience; IgA (polyclonal) from Jackson. Cells were stimulated for functional assessment overnight by culturing with 2.5 ng/mL phorbol myristate acetate and 1 μg/mL ionomycin in the presence of 1 μg/mL brefeldin A (BFA). Permeabilization for intracellular cytokine staining was performed using the eBioscience^™^ FoxP3/Transcription Factor Staining Buffer Set (Invitrogen^™^ 00–5523–00) according to the manufacturer protocol. Cell viability was determined using the Live/Dead Aqua Fixable Dead Cell Stain from Thermo Fisher Scientific. Lymphocytes were defined as clean, live, singlets based on clearly grouped populations, with subsets additionally defined by the use of internal controls and historical-determined expression of: CD20 for B cells, CD3 for T cells, CD11b for monocytes/macrophages, and NKG2a for NK cells. A threshold of 100 collected events in the parent population was used for all subset expression analyses. Analysis of flow data was performed with FlowJo v10.9.0.

### 16S isolation and analysis

DNA was isolated from 100 mg stool using the QIAsymphony PowerFecal Pro DNA Kit (Qiagen 938036) per the manufacturer’s protocol and sequenced using the Illumina MiSeq platform as previously described for the V4 region of the 16S rRNA gene (515F to 806R).^14^ Samples from individual animals were extracted and sequenced together to avoid batch effects. A custom R script was used to analyze Illumina FASTQ files. Paired-end FASTQ reads were filtered and processed using the dada2 package (v1.25.2) in R (v4.3.0). Reads were trimmed to 225 bp (forward) and 200 bp (reverse), then filtered to exclude sequences with degenerate bases (N), more than 2 expected errors (maxEE), or chimerism. Before filtering, 18.71 million reads were included in 66 samples with an average of 283, 521 reads per sample. After filtering and quality trimming, 11.63 million reads were included across all samples with an average of 176,171 reads per sample. Reads were binned into amplicon sequence variants (ASVs), then annotated with taxonomies using the SILVA taxonomic framework (release 138.1) at a 99% identity threshold and then analyzed using PhyloSeq (v1.46.0). ASVs identified as non-*Bacteria*, *Cyanobacteria*, or mitochondria (*Rickettsiales* mitochondria) were removed from further consideration, as were resultant genera at less than 3 % prevalence or phyla with no genera diversity.

### Ligilactobacillus salivarius qPCR

Forward primer For-Sal-3 (5′-GTCGTAACAAGGTAGCCGTAGGA-3′) and reverse primer Rev-Sal-1 (5′-TAAACAAAGTATTCGATAAATGTACAGGTT-3′) were used to detect *Ligilactobacillus salivarius* in extracted stool DNA via real-time quantitative PCR (RT-qPCR).^53^ Forward primer 16S_785F (5′-GGACTACGGATTAGATACCCTGGTAGTCC-3′) and reverse primer 16S_919R (5′-CTTGTGCGGGTCCCCGTCAAT-3′) were used to detect the 16S rRNA gene in the same samples via RT-qPCR. Samples were tested in duplicate. Amplification reaction mixtures contained 2 μL of template DNA, 12.5 μL of 2X PowerUp^™^ SYBR^™^ Green Master Mix (Applied Biosystems), 2 μL of each primer at 5 μM, and 6.5 μL of sterile nuclease-free water (Invitrogen). Sterile nuclease-free water was used for the no-template control in the place of template DNA. Assays were performed with a QuantStudio3 Real-Time PCR System (Applied Biosystems). Reaction mixtures were heated to 95 °C for 10 min prior to amplification. Standard thermal cycling conditions of 45 cycles of 98 °C for 10 s, 55 °C for 30 s, and 72 °C for 30 s were used. ΔCt values were calculated by subtracting the mean Ct of the 16S rRNA gene targeted reactions from the mean Ct of the *L. salivarius* targeted reactions.

### gDNA stimulation and flow cytometry

Glycerol stocks were used to inoculate 50 mL of MRS media (Teknova) then incubated overnight at 37 °C with 150 rpm shaking (n = 6 translocators, n = 4 non-translocators). DNA was extracted from non-translocator bacteria (isolated from RM stool) and translocator bacteria using the Qiagen Genomic-tip 500/G kit according to manufacturer instructions. DNA was concentrated or diluted to 465 ng/μL, to match the concentration of prepared Class C CpG oligonucleotides (InvivoGen). 20 μL of the prepared CpG oligonucleotides or bacterial DNA were added to 1 mL RPMI 1640 media supplemented with 10 % fetal bovine serum, 2 mM I-glutamine, and 1% penicillin/streptomycin (R10 media) (all from HyClone) with PBMCs from SIV-uninfected (n = 4) and SIV-infected RM (n = 2). Samples were then incubated for 48 h at 37 °C with 5% CO_2_. BFA was spiked in 16 h before end of stimulation to a final concentration of 1 μg/mL. Cells were stained and polychromatic flow cytometry run as described above. Antibodies against the listed antigens were used for staining at predetermined concentrations: CD20 (clone 2H7), CD3 (SP34–2), CD69 (FN50), IgG (G18–145), IgM (G20–127), and Ki67 (B56) from BD; HLA-DR (L243) from Biolegend. Cell viability was determined using the Live/Dead Aqua Fixable Dead Cell Stain from Thermo Fisher Scientific.

### Statistical analysis

Statistical analyses of growth assays, ELISAs, alpha diversity, qPCR, and flow cytometry data were performed using Prism version 9.3.1. AUC ratios from decitabine growth assays were compared between translocators and non-translocators at the tested concentrations of decitabine using one-tailed Welch’s *t*-test. 5-mC% results were compared using a one-tailed paired *t*-test. IL-6, sCD14, IFABP2, and Zonulin ELISA results were tested for significant changes by one-way ANOVA or mixed-effects model if missing values. Observed and alpha diversity measures were determined using the phyloseq package (v1.46.0) in R (v4.3.0), then compared for significant changes using one-way ANOVA. UniFrac values, weighted and unweighted, were determined using the phyloseq package then assessed for significant differences using Adonis analysis in the vegan package (v2.6–4) in R. The Analysis of Compositions of Microbiomes with Bias Correction (ANCOMBC) package (v2.4.0) in R was used to determine significantly altered ASVs between the baseline and treatment periods of the animal study using the ancombc function. One-way ANOVA was used to determine if there were significant changes in ΔCt values of *Ligilactobacillus salivarius* over the course of the animal study.

## Supplementary Material

1

## Data Availability

The data sets generated and analyzed during this study, including FASTQ files and metadata, are available in the NCBI Sequence Read Archive under accession no. PRJNA1032631.
