# Supplementary material for: Translocating bacteria in SIV infection are not stochastic and preferentially express cytosine methyltransferases
Source: Mucosal Immunol. Author manuscript; Available in PMC 2024 Oct 14. (PMC11471372; doi:10.1016/j.mucimm.2024.07.008)
Supplement: 1 [file NIHMS2016292-supplement-1.pdf]

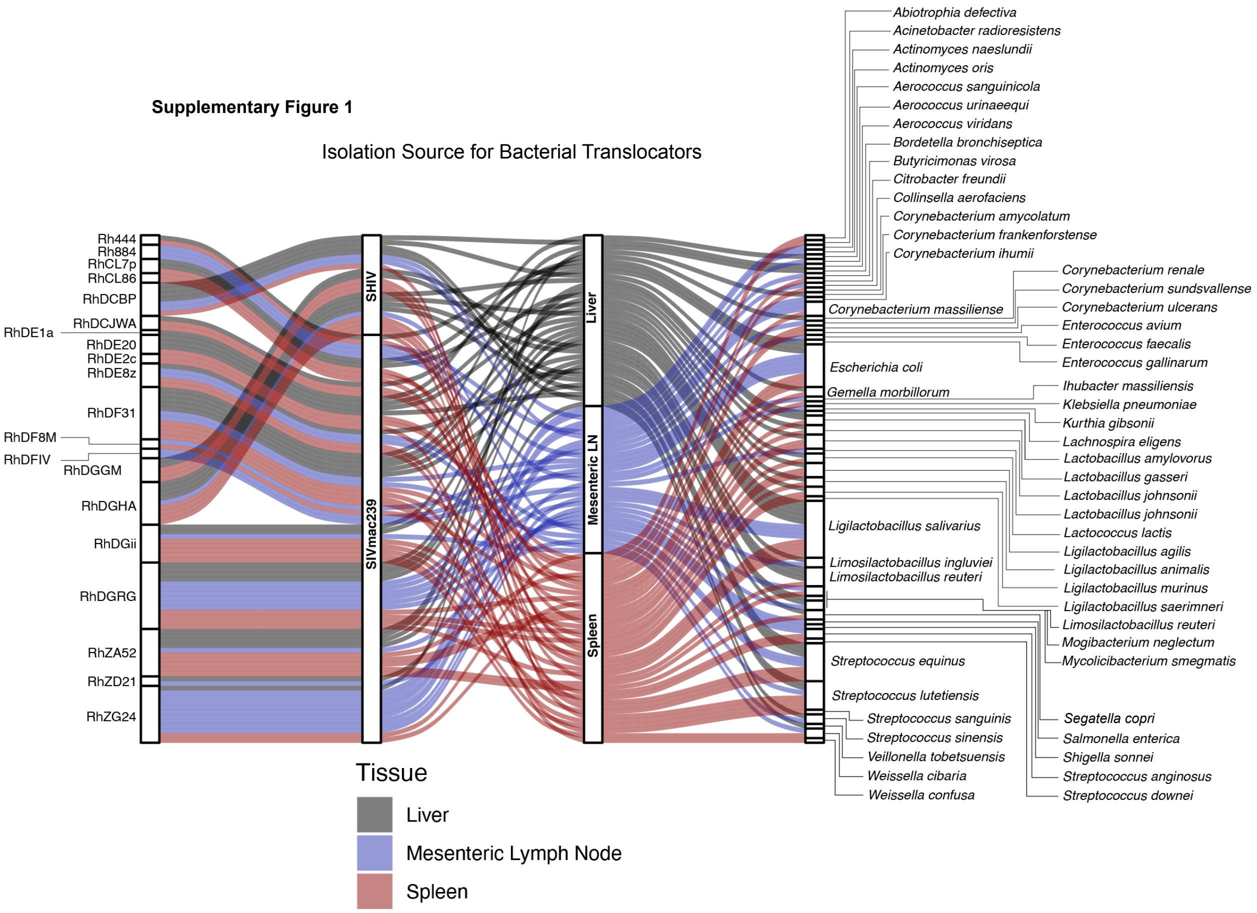

Supplementary Figure 1. Origins of Isolation of Translocating Bacteria. Alluvial plot showing the distribution of bacterial translocator isolates based on animal isolated from (n = 22), virus the animal was infected with, and the tissue of origin of the isolate.

Supplementary Figure 2

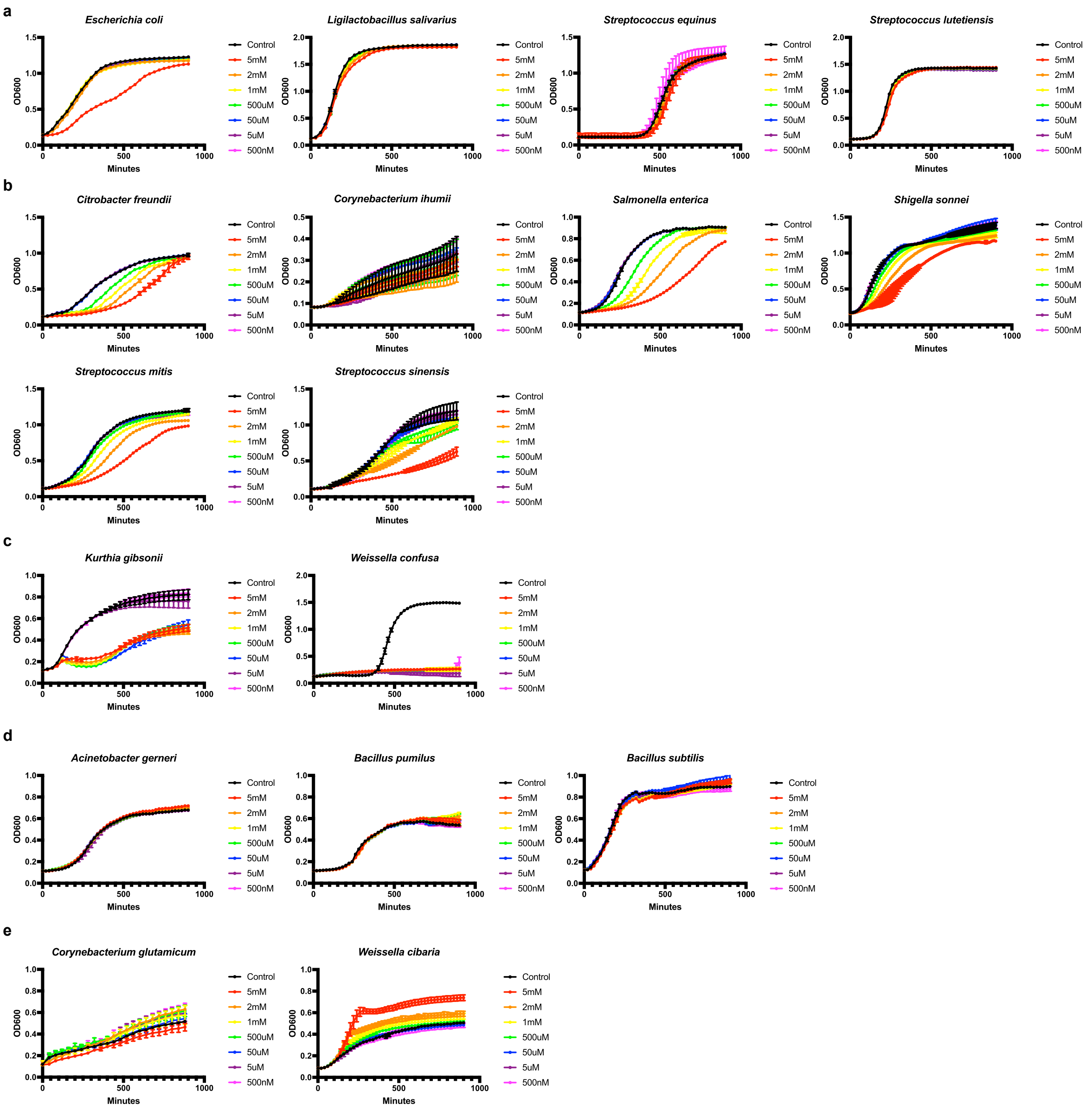

Supplementary Figure 2. *In Vitro* Decitabine Growth Assay Results. Growth curves of bacteria grown in the presence of decitabine at concentrations ranging from 5mM down to 500nM. Bars above and below represent the standard deviation of the data for samples run in triplicate. **a,** Growth curves of translocating bacterial taxa with non-responder phenotype. **b**, Growth curves of translocating bacterial taxa with dose-dependent responses. **c**, Growth curves of translocating bacterial taxa with high-sensitivity responder phenotype. **d**, Growth curves of non-translocating bacterial taxa with non-responder phenotype. **e**, Growth curves of non-translocating bacterial taxa with dose-dependent responder phenotype.

Supplementary Figure 3

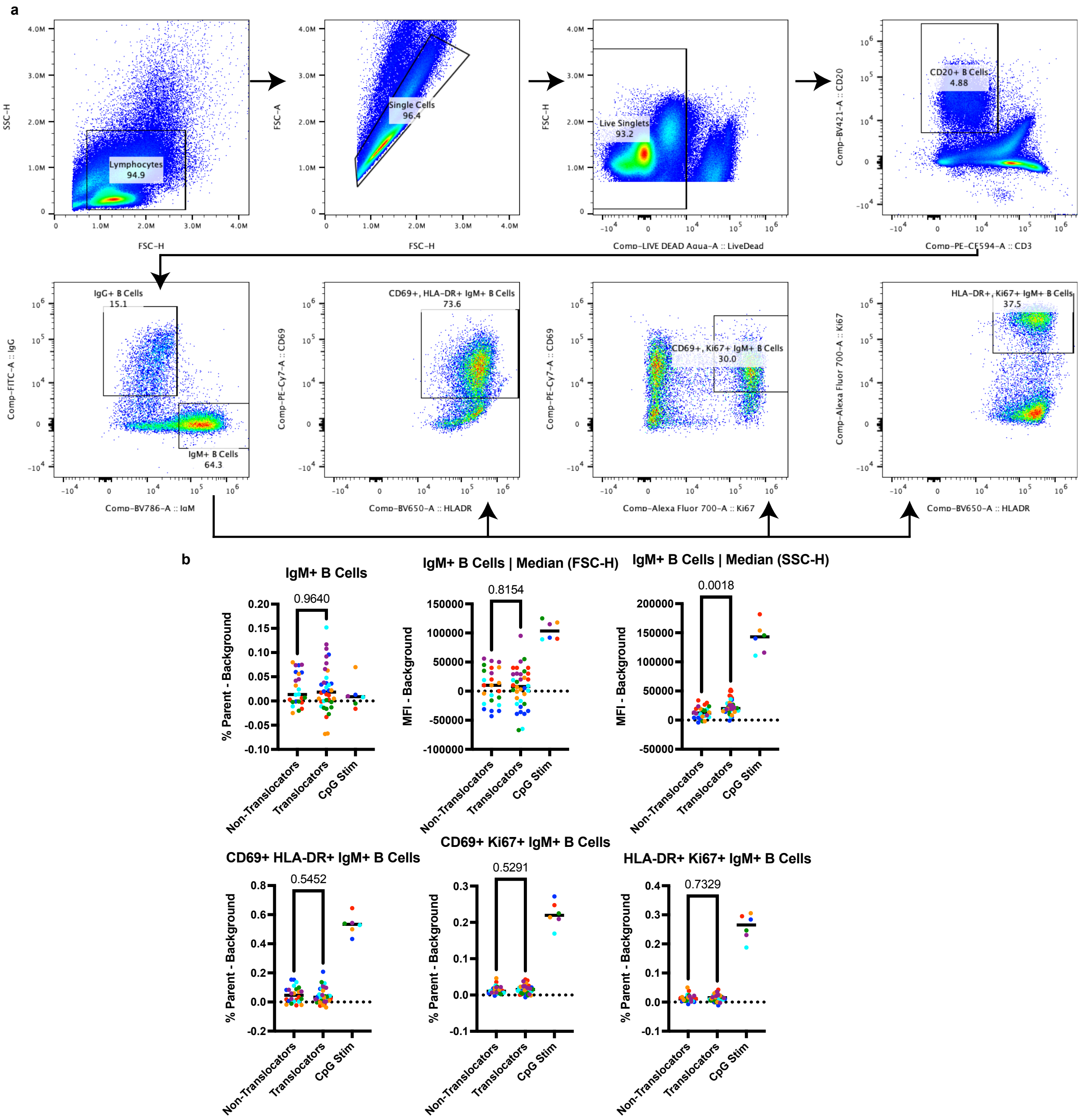

Supplementary Figure 3. ***In Vitro*** **Stimulation with Bacterial DNA. a**, Sample gating strategy used in bacterial gDNA stimulation experiments to assess activation of B-cells in response to translocator and non-translocator DNA. **b**, Comparison of immune cell populations after 48 hours of stimulation with gDNA isolated from translocators (n = 6) or non-translocators (n = 4), along with the effects of an unmethylated CpG oligonucleotide positive control (minus the results of unstimulated controls). Colors of dots indicate animals PBMCs were isolated from (n = 6). Non-translocator and translocator gDNA stimulated

populations were compared by unpaired t tests. Horizontal bars represent the median value.

Supplementary Figure 4

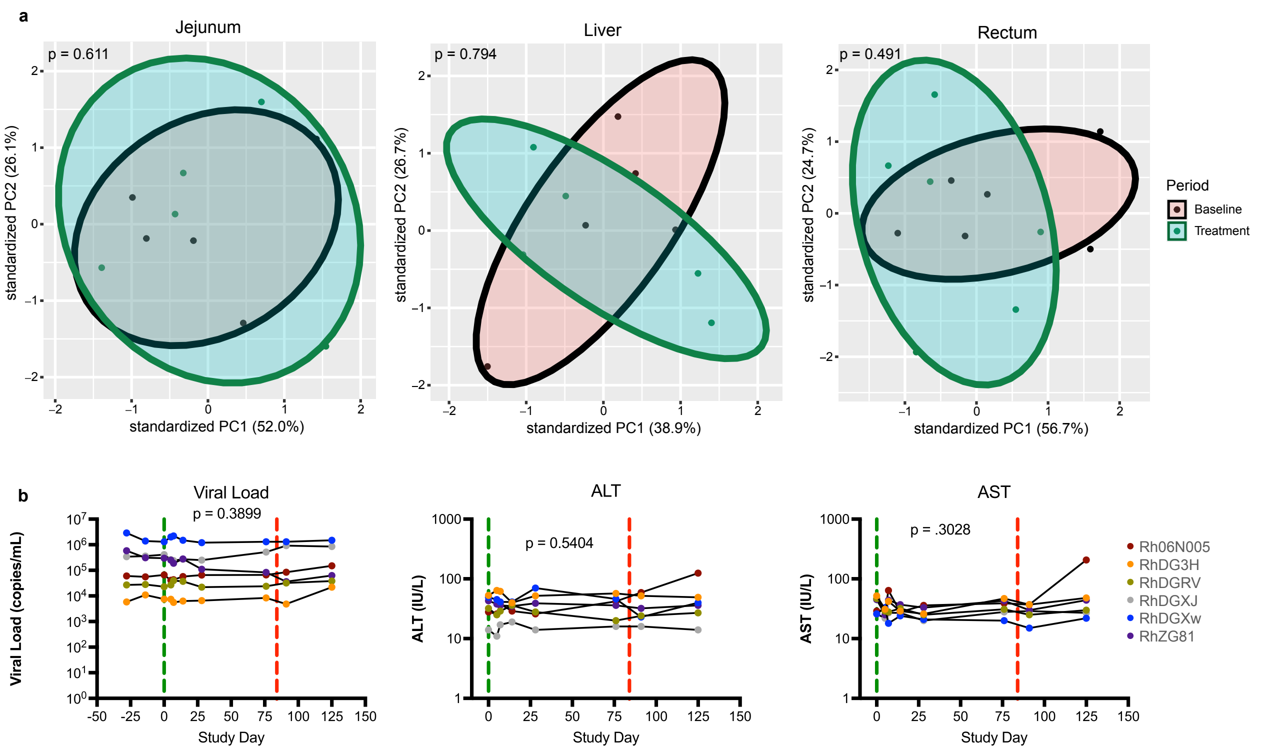

Supplementary Figure 4. *In Vivo* Blood Markers and Immune Cell Responses to Decitabine. a, Principal Component Analysis (PCA) plots of immune cell population percentages from jejunal, liver, and rectal biopsies taken at D0, before treatment, and D76 into decitabine treatment of RM (n = 6). Considers phenotypic markers of B cells, macrophages, and NK cells and phenotypic and functional markers of T cells. The 95% confidence interval is represented by the ellipse for each sample group. Adonis was used to test for significant differences in jejunal, liver, and rectal biopsy immune populations (p = 0.611, p = 0.794, and p = 0.491, respectively). **b,** Plasma viral load and alanine transaminase (ALT) and aspartate aminotransferase (AST) concentrations in blood of decitabine treated RM (n = 6). Changes assessed for significance by one-way ANOVA.

Supplementary Table 1

Animal	Virus	CD4 Count at Necropsy	Viral Load at Necropsy	Liver	Mesenteric Lymph Node	Spleen
RH444	SIVmac239	(Undetermined)	140,000	1	0	1
Rh884	SIVmac239	629	1,670,000	0	3	0
RhCL7p	SIVmac239	146	3,000,000	2	0	1
RhCL86	SIVmac239	58	1,900,000	0	0	2
RhDCBC	SIVmac239	71	700,000	0	1	1
RhDCBP	SHIV	176.58	2,100,000	4	2	1
RhDCJWA	SIVmac239	27	2,100,000	1	0	2
RhDE1a	SIVmac239	362	490,000	1	0	0
RhDE20	SIVmac239	742	87,000	3	0	1
RhDE2c	SIVmac239	619	420,000	0	0	2
RhDE8z	SIVmac239	283.05	4,000,000	1	2	2
RhDF31	SIVmac239	(Undetermined)	1,300,000	5	2	4
RhDF52	SIVmac239	(Undetermined)	3,210,000	1	0	0
RhDF8M	SIVmac239	(Undetermined)	(Undetermined)	0	1	1
RhDFIV	SIVmac239	661.23	810,000	0	2	0
RhDGGM	SHIV	563.2	300,000	2	0	3
RhDGHA	SHIV	964.6	300,000	4	1	4
RhDGii	SIVmac239	354.816	(Undetermined)	2	1	5
RhDGRG	SIVmac239	1036.796	(Undetermined)	4	6	4
RhZA52	SIVmac239	133	1,300,000	4	1	5
RhZD21	SIVmac239	30.222	840,000	1	1	0
RhZG24	SIVmac239	26	550,000	1	9	2

Supplementary Table 1. Animals Used in Isolation of Translocators. Information on animals used for translocator isolation at the time of necropsy, including virus infected with, CD4 count, viral load, and the number of bacterial isolates from the animal along with the tissues isolated from.
